# Dynamic variations in salinity and potassium grade of a potassium-rich brine deposit in Lop Nor basin, China

**DOI:** 10.1038/s41598-021-82958-y

**Published:** 2021-02-08

**Authors:** Lichun Ma, Kai Wang, Yu Zhang, Qingfeng Tang, Hui Yan

**Affiliations:** 1 MNR Key Laboratory of Metallogeny and Mineral Assessment, Institute of Mineral Resources, Chinese Academy of Geological Science, Beijing, 100037 China; 2 China University of Geosciences(Beijing), Beijing, 100083 China; 3 Beijing Centre for Physical and Chemical Analysis, Beijing, 100089 China; 4 SDIC Xinjiang Lop Nor Potash Co., Ltd., Hami, 839000 China

**Keywords:** Geochemistry, Geology, Mineralogy

## Abstract

The Quaternary Lop Nor playa is the largest production base of potassium sulfate in the world. It has a mining history of more than 10 years, and its share in the Chinese potassium sulfate market is about 50% to-date. In this basin, the high-salinity potassium-rich brines are mainly contained in Middle Pleistocene–Holocene glauberite strata. Based on the monitoring of the underground brine table and geochemical analysis, this study reveals variations in the underground brine table and potassium-bearing grade before and after large-scale mining in the Lop Nor potash deposit. The results showed that the underground brine table and potassium sulfate grade decreased by varying degrees over sub-mineral areas after large-scale mining. The underground brine table declined by 8.5 m, on average, in the Luobei depression, by 6.4 m in the Tenglong platform and by 1.9 m in the Xinqing platform. However, the potassium-bearing grade showed the different trend. The Tenglong platform had the largest decline with average decreases in layers W_1_, W_2_ and W_3_ of 18.2%, 13.0% and 24.8%, respectively. In the Xinqing platform, the average decrease in layersW_2_ and W_3_ were 17.4% and 16.0% respectively. The Luobei depression decreases were relatively small (W_1_, W_2_ and W_3_ decreased 4.3%, 4.2% and 3.1%, respectively). This research provides a theoretical basis for the rational development and sustainable use of the potassium-rich brines in the Lop Nor basin.

## Introduction

The Lop Nor playa is located in the eastern Tarim Basin (Xinjiang, China) and is a famous Quaternary inland salt lake that is also the largest single liquid deposit of potassium sulfate in the world. The high-salinity potassium-rich brine is mainly contained in Middle Pleistocene–Holocene glauberite strata. The SDIC Xinjiang Lop Nor Potash Co., Ltd. (abbreviate: SLNP), founded in 2000, has the exploration and mining rights for the potash deposit. In 2003, SLNP carried out mining tests in the hinterland of Lop Nor basin. At the end of 2005, it reached an annual production capacity of 100,000 tons of potassium sulfate and in 2012, it achieved an annual output of 1.37 million tons. In 2017, it reached 1.5 million tons of production capacity. At present, its share in the Chinese potassium sulfate market is about 50%.

Since the discovery of potassium-rich brine deposits in the Lop Nor basin, there has been a lot of research in the area. In the past 30 years, researchers have made great progress in metallogenic conditions and sedimentary environment analysis^1–6^, ore deposit characteristic and genesis^2,7,8^, geochemical characteristics of the potassium-rich brine reservoir body^2,9^ and mining process and technology^10–13^. However, there are few studies on the dynamic variations in the geochemistry of potassium-rich brine deposits in Lop Nor basin^14^. In 2006, 2009 and 2010, general exploration of all three sub-mineral areas (Luobei depression, and Tenglong and Xinqing platforms) in Lop Nor basin was carried out. Exploration obtained a large amount of drilling and brine chemistry data, which laid the foundation for this research. Based on the geochemical analysis of different ore horizons from the early general exploration reports^15–17^ and the brine samples and chemical data collected in the mining area in 2017, this study conducted a comprehensive comparison. It revealed the temporal and spatial variations of the brine mineralization and potassium grade in different ore horizons before and after large-scale mining in Lop Nor basin. Our results provide a theoretical basis for the rational development and sustainable use of potassium-rich brines in Lop Nor basin.

## Regional geological background

**Figure 1:**
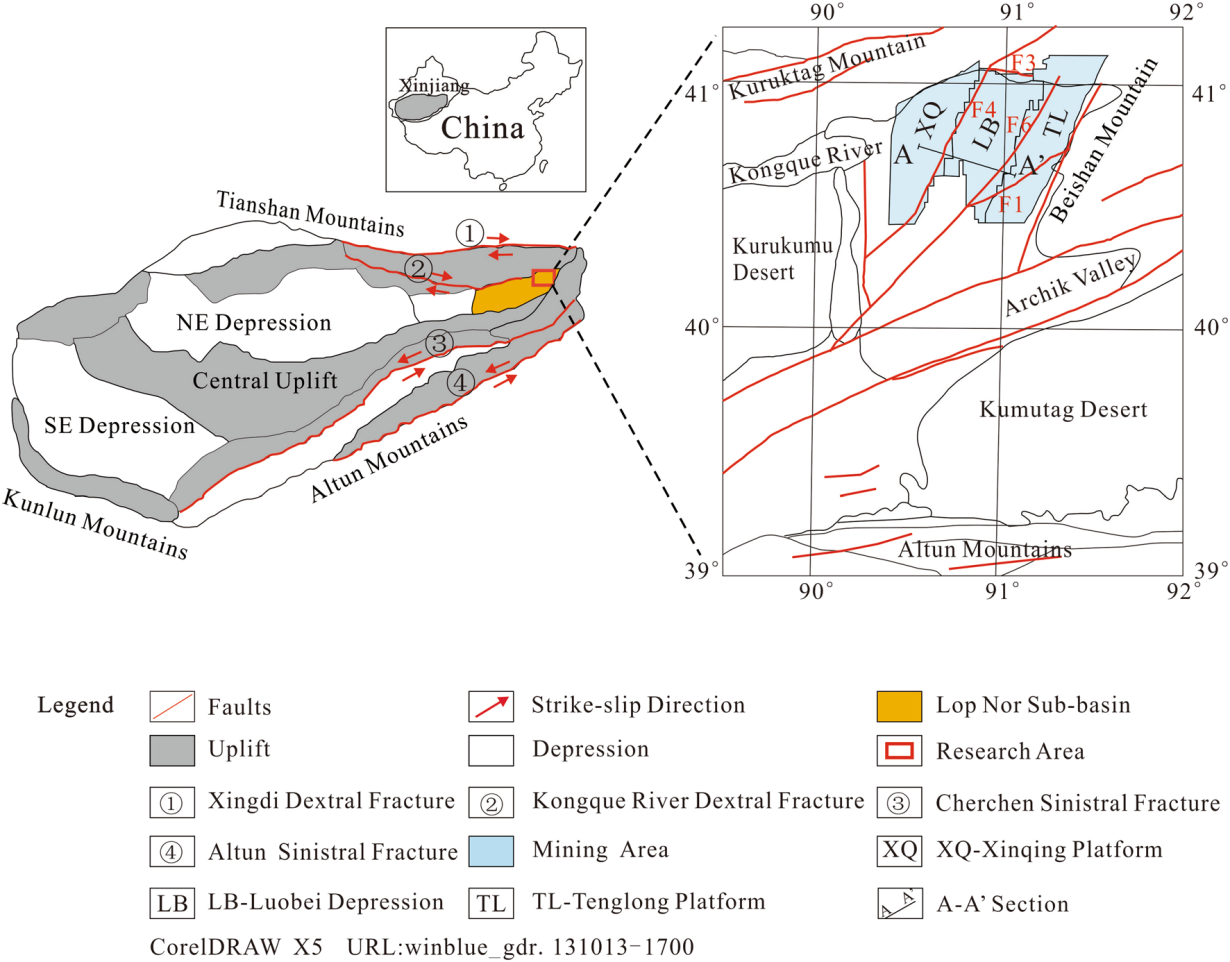
Simplified geological map of the Lop Nor area in Xinjiang Province, China.

Lop Nor is located at the intersection of the Altun and Beishan tectonic belts in the eastern part of the Tarim platform. The northern and southern basin boundary is controlled by the Kongque River fault and the Altun fault, respectively^13^ (Fig. 1). The regional tectonic environment is complex, the basement is fractured and the inheritance and neotectonic activity are strong. The fault structure generally restricts the formation and development of Lop Nor Salt Lake^2^. Since the end of the Neogene, the Lop Nor area has been controlled by tectonic movement^5^; it began to settle and gradually developed into the lowest depression of the basin. Glacial meltwater, originating from the Tianshan, Kunlun and Altun Mountains, eventually gathered in the Lop Nor basin^13^; the water area once reached 20,000 km^2^. Therefore, the Lop Nor Lake played an important role as a catchment center of the whole Tarim basin throughout the Quaternary by accumulating a large amount of salt in the basin^13^. At present, the lake has completely dried up with a salt cover spanning 10,000 km^2^; the salt deposit is more than 200 m thick. Potassium-rich brines are found in these Quaternary salt strata in the northern Lop Nor basin.

The potassium-rich brine deposit consists of three sub-mineral areas from west to east, including the Xinqing platform, Luobei depression and Tenglong platform. The boundary between the sub-mines and the brine reservoir is mainly controlled by faults. According to the fault direction, the system can be roughly divided into three groups: faults in the NNE, faults near the EW and faults in the NEE. The faults in the NEE are the most developed and include the F4 fault, which is between the Xinqing mining area and the Luobei depression, and the F6 fault, which is the boundary of the Luobei depression and the Tenglong platform^14^. The F1 fault is a regional compression–torsion fault, which passes through the Tenglong mining area and cuts it into two parts, north and south^14^ (Fig. 1).

Luobei depression is located in the middle of the whole mining area. It is about 60 km long from north to south, 32.5 km wide from east to west, and has an area of about 1534 km^2^ (Fig. 1). The overall terrain is lower than the Tenglong and Xinqing platforms to the east and west with an average altitude of about 780 m, and the surface is covered by salt crusts. The thickness of the salt strata ranges from about 30–200 m, and the average thickness is about 100 m. The salt system tends to thicken from south to north and the thickness also gradually increases from west to east^15^.

The Tenglong platform is about 70–90 km long from north to south and about 20–25 km wide from east to west with an area of about 1623 km^2^ (Fig. 1). The surface is mainly a Yadan landform and the terrain is higher than the Luobei depression, with a maximum altitude of 790 m and a minimum altitude of 780 m (average = 785 m). The thickness of the salt-bearing strata in the northern part of the Tenglong platform is generally 30–50 m (average = 36 m) with a maximum thickness of 69.6 m. The thickness gradually increases from north (12 m) to south (60 m) and from east (20 m) to west (60 m). The thickness of the salt-bearing strata is generally 5–20 m south of the F1 fault, and gradually increases from south to north (4–12 m) and decreases from east to west with a thick middle area^16^.

Xinqing platform is about 60–80 km long (north–south) and 10–20 km wide (east–west) with an area of about 1447 km^2^ (Fig. 1). The surface is mainly a Yadan landform and the terrain is relatively high (highest altitude = 795 m, lowest altitude = 780 m) with an average elevation of 789 m. The accumulated thickness of the salt-bearing strata in the Xinqing platform is about 30–50 m. It gradually thickens from south to north, as well as west to east^17^.

The whole Lop Nor playa currently has no surface water system. It mainly receives bedrock fissure water and groundwater recharge from Kuluktag Mountain, Beishan Mountain and the Altun Mountains. It also receives lateral recharge from the eastern Archik Valley, as well as the western Kongque River and Tarim River dry deltas (Fig. 1). Down the regional hydrologic gradient, as groundwater moves from the outer fringes to the center of the playa, its salinity gradually increase, reaching a maximum of roughly 350 g/L.

## Methods

### Sampling and analysis

In 2003, SLNP conducted a mining test in the Lop Nor playa. The underground brine was pumped to the surface through a shaft system, and then transported through a brine channel to the solar pond for salt drying and classification. Following an economic feasibility analysis, the current four-layer brine (top to bottom: W_1_, W_2_, W_3_ and W_4_) was mainly mined within 90 m. However, the amount mined early on was relatively small. At the end of 2005, the production capacity was only 100,000 tons (K_2_SO_4_) per year. The potassium sulfate project goal of 1.2 million tons per year started ran until November 2008. In November 2011, the monthly design production capacity was reached. This meant that large-scale brine mining had not been carried out in the Lop Nor playa before 2011. To find the potash reserves in the mining area, SLNP carried out general exploration operations in July 2006, March 2009 and August 2010 in the Luobei depression, Tenglong platform and Xinqing platform, respectively. The exploration data provided the basis for the study of geochemical variations before and after large-scale mining in the Lop Nor basin.

The chemistry analysis from the three sub-mines areas used in this study was derived from the general exploration reports from 2006, 2009 and 2010^15–17^. Data were generated from 88 brine samples collected in July–August 2017 through observation holes in the mine area. Two bottles (500 mL each) were used to sample each observation hole. The brine depth and density were measured on site and the bottles were sealed quickly after measurements to prevent the brine from evaporating or leaking during transport. GPS data were used for geolocation and elevation measurements.

The brine samples taken in 2017 were sent to the National Geological Experiment and Test Center (Chinese Academy of Geological Sciences) for the analysis of major components (Na^+^, K^+^, Mg^2+^, Ca^2+^, Cl^−^, SO_4_^2−^, CO_3_^2−^ and HCO_3_^−^) and trace elements (Li^+^, B^3+^, Br^−^, I^−^, Rb^+^, Cs^+^ and Sr^2+^) in which K^+^, Na^+^, Mg^2+^, Ca^2+^, Li^+^, B^3+^, Rb^+^, Cs^+^ and Sr^2+^ were determined by atomic absorption spectrophotometry (RSD < 2%), Cl^−^ and SO_4_^2−^ were determined by ion-chromatography (RSD < 2%). Titrimetric methods were used for the determination of Br^−^, I^−^, CO_3_^2−^ and HCO_3_^−^ (RSD < 5%).

### Mapping methods

According to the brine chemistry analysis and the groundwater table data, a spatial distribution map of brine geochemistry and groundwater drawdown in different ore horizons of the three sub-mining areas were drawn by Kriging interpolation in the software Surfer.

## Results and discussion

**Figure 2:**
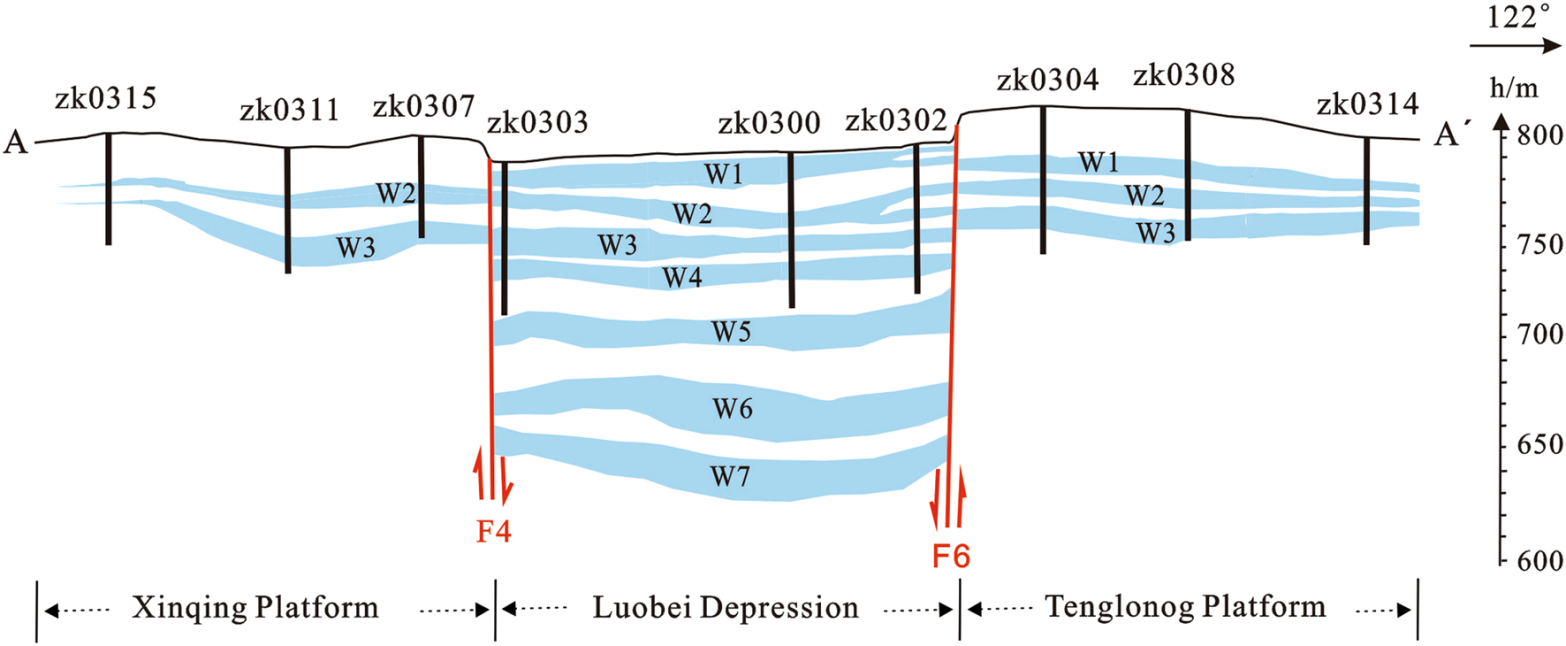
Cross-section of the potassium-rich brine deposit in Lop Nor basin.

Lop Nor is a typical liquid deposit of potassium sulfate. Based on the Valyashko classification system^18^, the water chemistry type is mainly a magnesium sulfate subtype and, secondly, a sodium sulfate subtype. The current brine salinity range is about 226–393 g/L and the average grade of KCl is about 1.36%. The brine reservoir is mainly located in the glauberite layer, then the coarse clastic layer and a very small amount is in the halite and gypsum layer. The number of potassium-rich brines layers is different in each sub-mineral area, which is controlled by the structure and fault system of the mining area. There are seven brine layers (W_1_–W_7_) within 250 m depth in the Luobei depression, including one layer of phreatic water and six layers of confined water (Fig. 2). The Xinqing mining area has two layers of confined water (Fig. 2). The Tenglong mining area has three potassium-rich brine layers, including a phreatic water layer and two confined water layers (Fig. 2). The Location of cross-section A–A′ is shown in Fig. 1.

According to analyses of principal and trace elements from the brine samples collected in 2017, the available elements in the brine of the Lop Nor playa included elemental B in addition to K, and the content of B_2_O_3_ in the brine varied from 277.3 to 755.6 mg/L, which is greater than the comprehensive utilization grade (150 mg/L). However, B has not been exploited yet, so it will not be discussed in this study.

### Luobei depression

**Figure 3:**
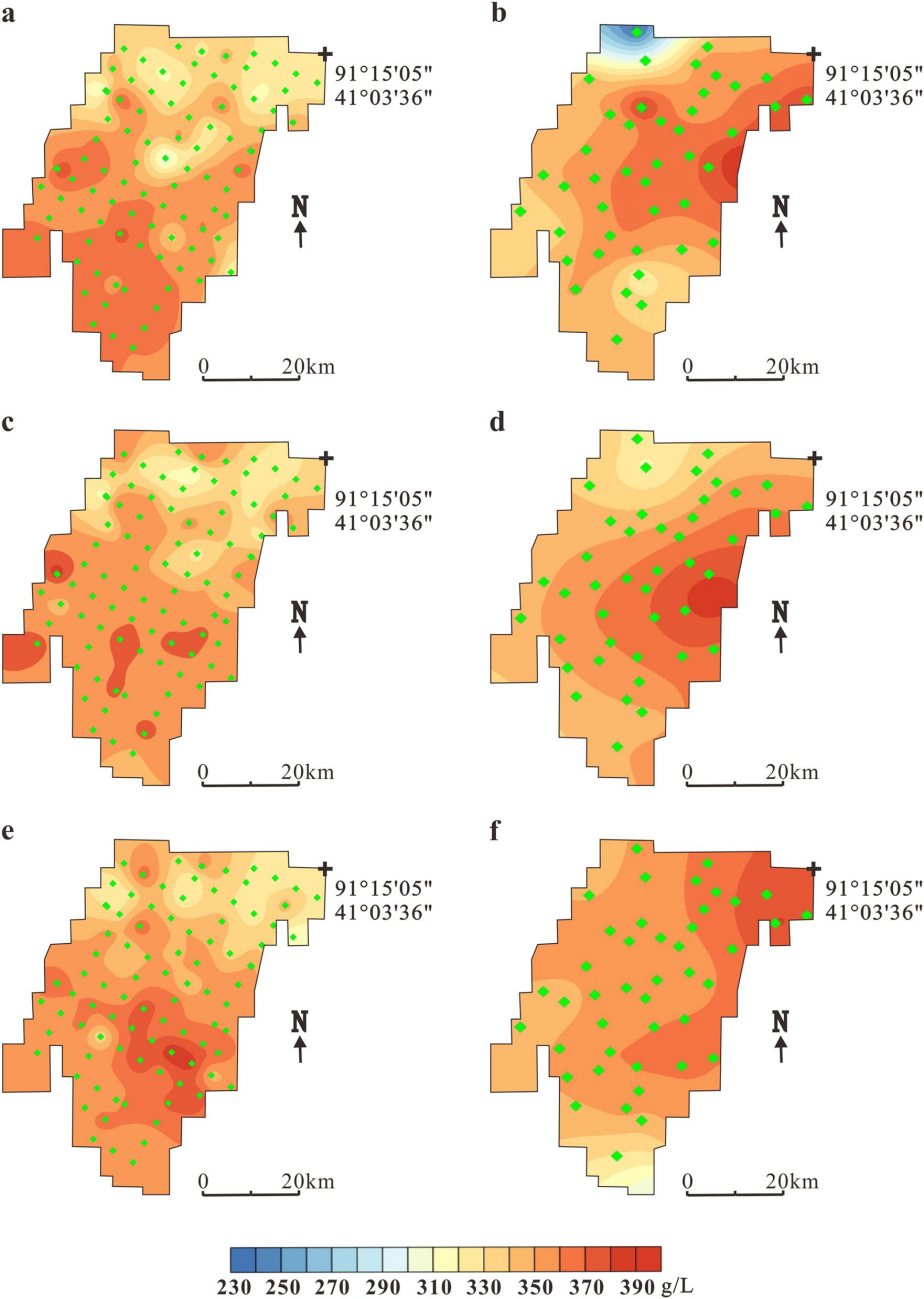
Temporal and spatial variations in the salinity of different brine layers in Luobei depression. (**a**) Layer W_1_ in 2006, (**b**) Layer W_1_ in 2017, (**c**) Layer W_2_ in 2006, (**d**) Layer W_2_ in 2017, (**e**) Layer W_3_ in 2006 and (**f**) Layer W_3_ in 2017. The green dots indicate observation holes locations.

Luobei depression is the main storage area for potassium-rich brine. Through exploration in 2006, it was found that the KCl (122b + 333) specific yield reserve in the elevation range of 628–786 m was 8384.84 million tons^15^, and long-term observation holes were established for monitoring variations in chemical and physical properties and the water table of the underground brine in three main ore layers (W1, W2, W3). In 2006, the burial depth of the ore layer W_1_, W_2_, W_3_ and W_4_ was 1.7–2.3 m, 20–40 m, 40–60 m and 55–75 m respectively. After 11 years of mining, the brine table of layer W_1_ decreased by 8.5 m on average. Figure 3 shows the comparison of the salinity in three ore layers from 2006 and 2017, including the temporal and spatial variations in brine salinity before and after 11 years of mining in the Luobei depression.

In 2006, the salinity of the ore layer W_1_ in the Luobei depression was 295.9–387.8 g/L and the average salinity was 348.8 g/L^15^ (Fig. 3a). The low-salinity area is located in the northern part of the mining area with higher values located in the south. After 11 years of mining, the salinity ranged from 234.7 to 387.4 g/L and was significantly reduced in the northern and southwestern regions, which may have been caused by the recharge of freshwater from the northern piedmont zone and the western dry delta. The high-salinity area is offset to the northeast, which means that the underground brine formed a new flow field and the concentration center moved to the Tenglong platform (Fig. 3b). Ore layers W_2_ and W_1_ showed the same trends; in 2006, the salinity in W_2_ was 310.3–387.6 g/L, but in 2017, the salinity was changed to 316.9–367.7 g/L. The concentration center also migrated from the south–central part of the mining area to the east. After years of mining, the brine in the north, west and southwest is obviously depleted (Fig. 3c, d). The salinity of layer W_3_ in the south was also higher than in the north. The highest value was 389.4 g/L and the lowest value was 307.2 g/L with an average salinity of 352.1 g/L (Fig. 3e). After mining, the salinity varied between 344.5–380.6 g/L and the high-salinity area moved to the north where brine salinity increased obviously. This may be because of high-salinity brine recharge from the eastern and western platforms after the formation of new flow fields. As the depth increased, W_3_ was not significantly affected by freshwater from the northern piedmont zone and western deltas (Fig. 3f).

**Figure 4:**
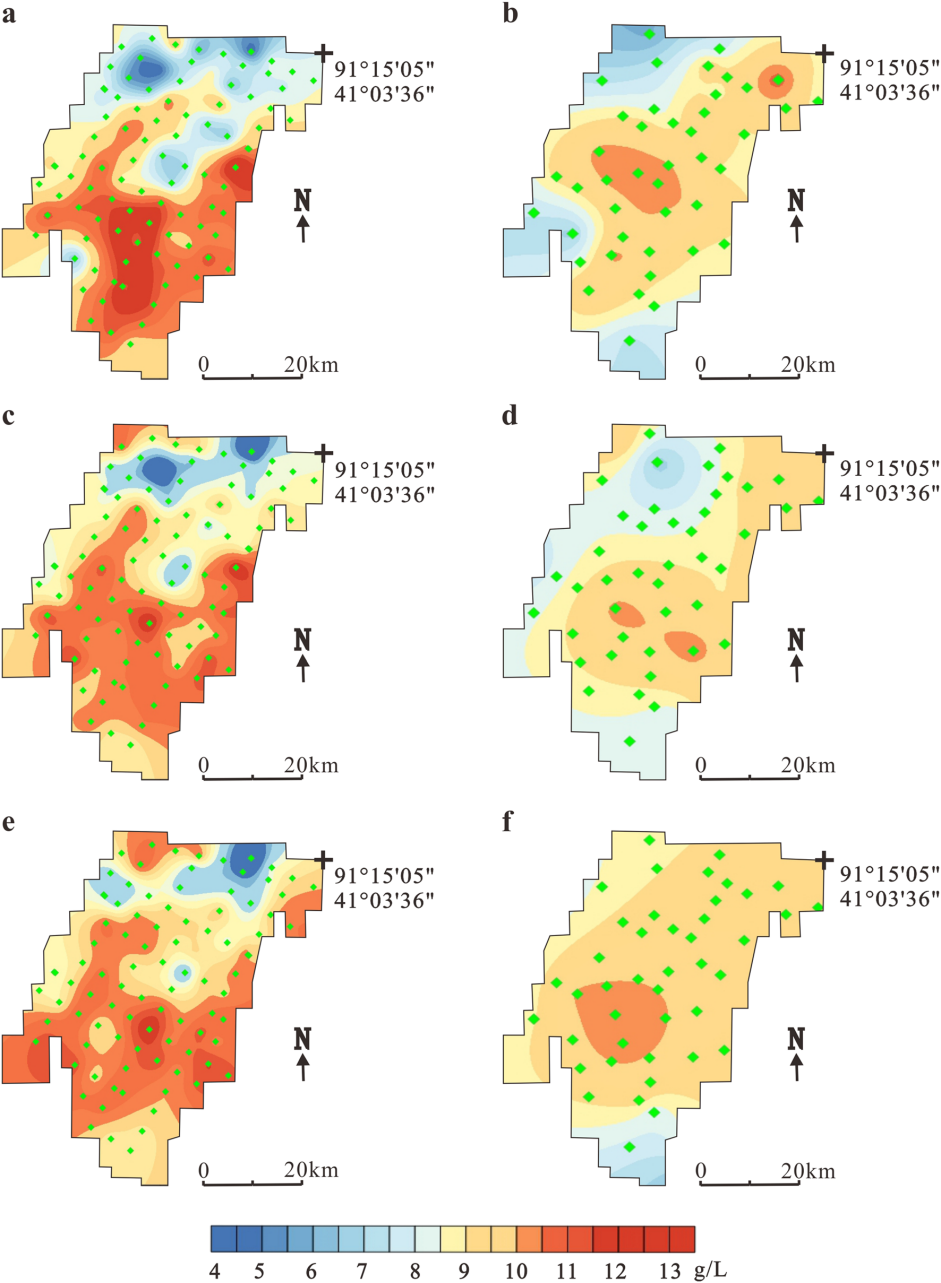
Temporal and spatial variations in K^+^ content of different brine layers in Luobei depression. (**a**) Layer W_1_ in 2006, (**b**) Layer W_1_ in 2017, (**c**) Layer W_2_ in 2006, (**d**) Layer W_2_ in 2017, (**e**) Layer W_3_ in 2006 and (**f**) Layer W_3_ in 2017. The green dots indicate observation holes locations.

The spatial distribution of K^+^ content in three ore layers in 2006 and 2017 is shown in Fig. 4. In 2006, the K^+^ content of W_1_ in the Luobei depression increased from 4.41 to 13.4 g/L with an average content of 9.4 g/L. The main high-value area is located in the southern part of the mining area. The K^+^ content in the northern part of the mining area was relatively low (5.5–8 g/L), which may be related to the recharge of the bedrock fissure water from Kuluktag Mountain in the north(Fig. 4a). After 11 years of mining, in 2017, the K^+^ content changed to 5.9–10.9 g/L (average = 9.0 g/L), which was an average decrease of 4.3%. The main high-value area shrunk and was located in the central and northeastern parts of the mining area (Fig. 4b).

In 2006, the K^+^ content of W_2_ was 6.9–12.3 g/L with an average of 9.5 g/L. The K^+^ distribution was similar to W_1_. The high-value area is mainly located in the southern part of the mining area, and the low-value area is located in the north (Fig. 4c). After mining, the K^+^ content was 6.67–10.3 g/L (average = 9.1 g/L), which was a decline of about 4.2%, and the high-value area shrunk from the south–central part of the mining area to the southeastern part (Fig. 4d). Before mining ore layer W_3_, the distribution of K^+^ was similar to W_1_ and W_2_ in that it was also characterized by a low-value north and a high-value south. The K^+^ content varied from 6.9 to 12.4 g/L with an average of 9.8 g/L (Fig. 4e). After mining, the K^+^ content generally changed to 8.2–10.4 g/L and the average content decreased from 9.8 to 9.5 g/L, a decline of 3.1% (Fig. 4f).

### Tenglong platform

The Tenglong mining area exposed three layers of potassium-rich brine, including a phreatic water layer and two confined water layers. The phreatic brine ore body is the main ore body of the Tenglong mining area. It is buried at shallow depth with the water table at about 0.7–4.0 m. However, the phreatic brine ore body is bounded by a fracture (F1) and only present in the northern part of the Tenglong mining area. The burial depth of the confined brine layer W_2_, W_3_ was about 15–30 m, 20-40 m respectively.

**Figure 5:**
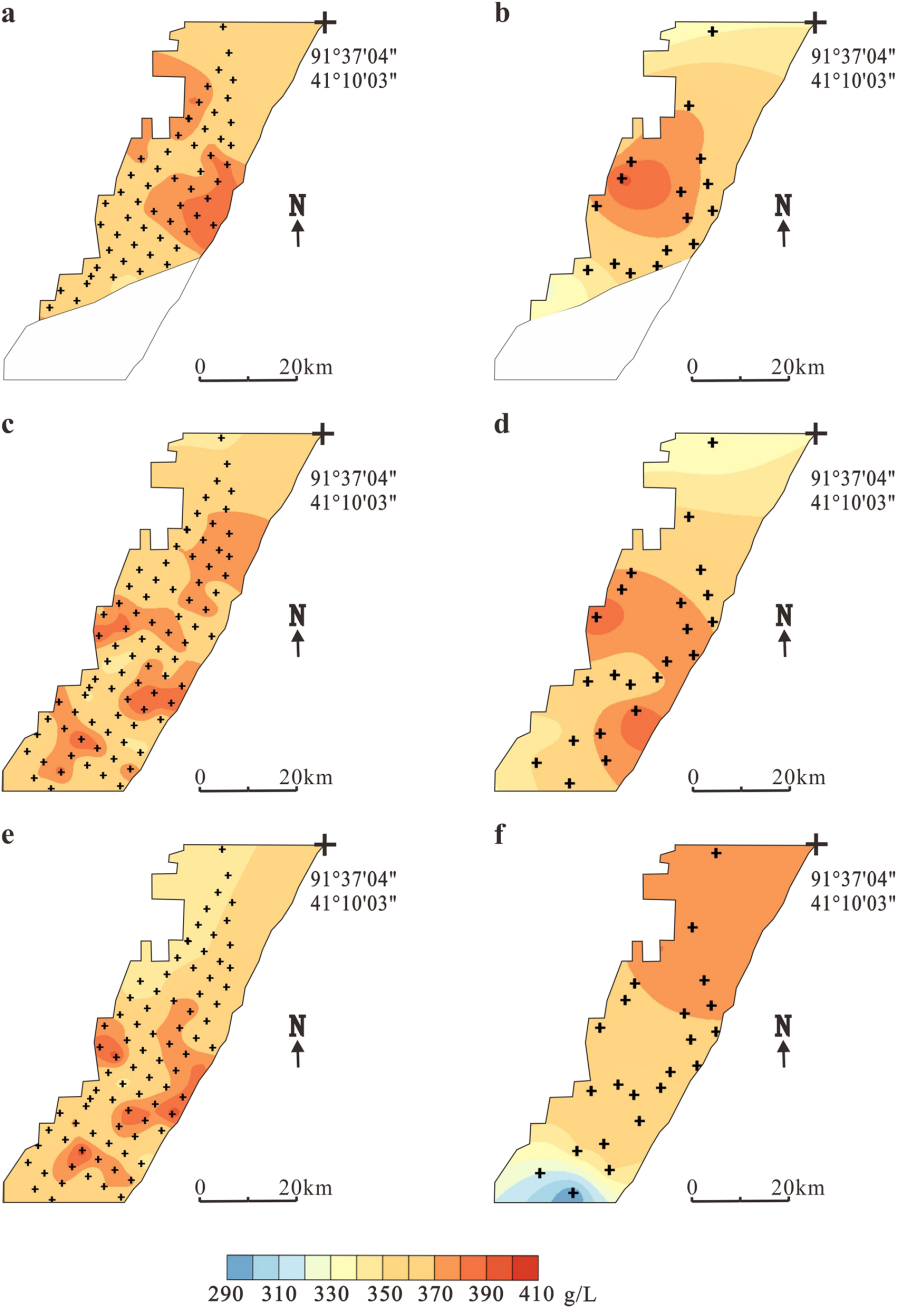
Temporal and spatial variations in salinity of different brine layers in Tenglong platform. (**a**) Layer W_1_ in 2009, (**b**) Layer W_1_ in 2017, (**c**) Layer W_2_ in 2009, (**d**) Layer W_2_ in 2017, (**e**) Layer W_3_ in 2009 and (**f**) Layer W_3_ in 2017. The black crosses indicate observation holes locations.

In March 2009, exploration determined a specific yield reserve of KCl (122b + 333) of 26.53 million tons at 707–787 m elevation^16^. Figure 5 shows a comparison of the salinity of three ore layers in 2009 and 2017, specifically the temporal and spatial variations in salinity before and after large-scale mining in the Tenglong mining area.

The salinity of horizon W_1_ in the Tenglong mining area in 2009 was 344.5–390.1 g/L with an average value of 365.3 g/L^16^ (Fig. 5a). The high-salinity area is mainly located in the middle of the mining area and the low-value area is located in the southwest and north. After 8 years of mining, the salinity trends were basically the same, the values changed from 329.2 to 379.4 g/L (Fig. 5b). In 2009, the salinity of horizon W_2_ changed from 342.7 to 391.5 g/L (average = 366.9 g/L). The high-value area was distributed in the east–central and south areas, and low salinity is mainly concentrated in the northern part of the mining area (Fig. 5c). In 2017, after mining, the salinity range changed to 331.2–387.7 g/L and the southeastern part of the mining area was obviously diluted (Fig. 5d). Horizons W_3_, W_1_ and W_2_ showed a consistent distribution trend before and after mining. The post-mining salinity range was reduced from 343.9–397.7 to 290.9–353.7 g/L, a decline of 8.4% (Fig. 5e, f).

**Figure 6:**
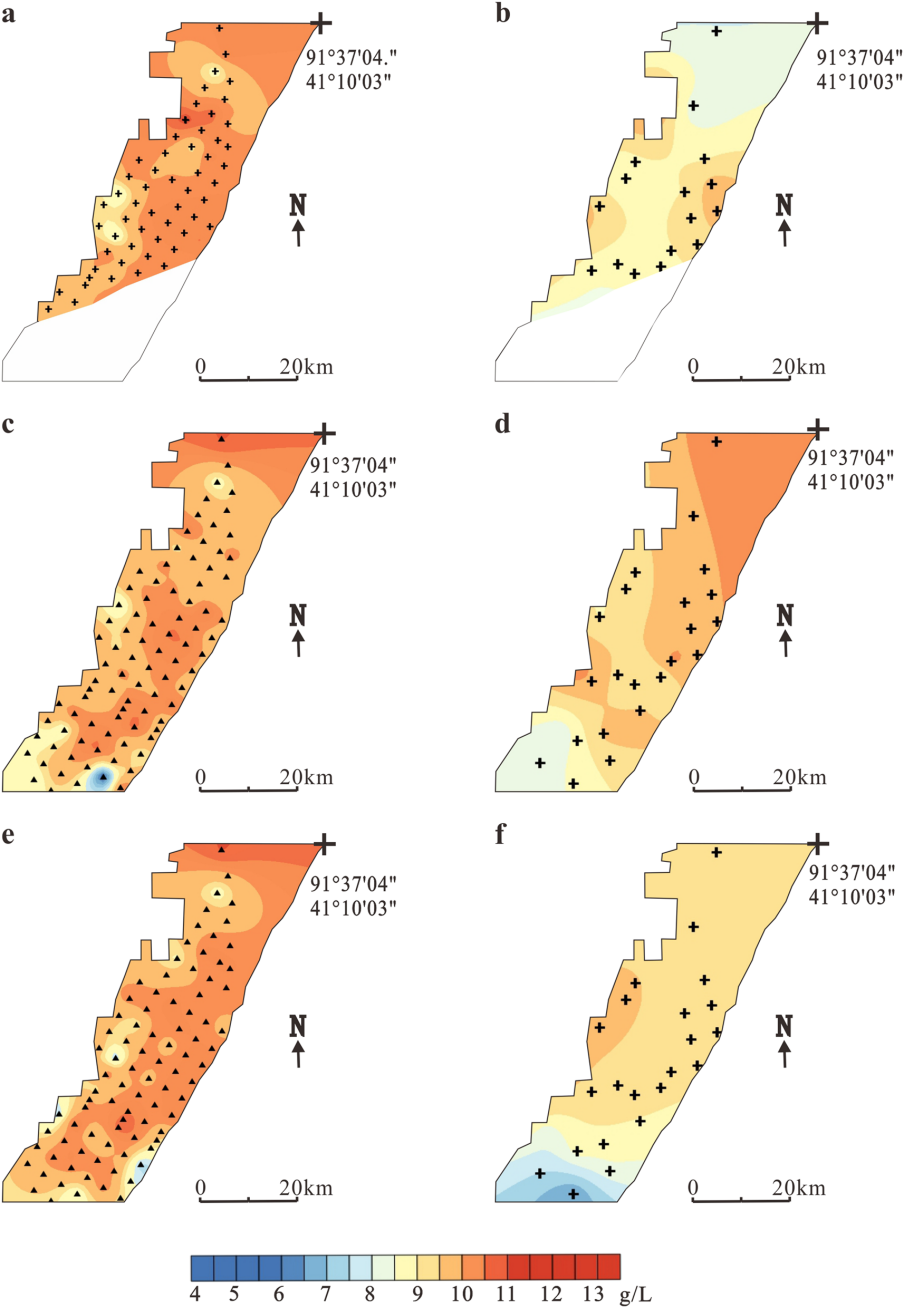
Temporal and spatial variations in K + content of different brine layers in Tenglong platform. (**a**) Layer W_1_ in 2009, (**b**) Layer W_1_ in 2017, (**c**) Layer W_2_ in 2009, (**d**) Layer W_2_ in 2017, (**e**) Layer W_3_ in 2009 and (**f**) Layer W_3_ in 2017. The black crosses indicate observation holes locations.

Figure 6 compares the spatial distribution of K^+^ content before and after mining in the Tenglong platform. The K^+^ content in W_1_ in 2009 changed from 8.9 to 12.9 g/L with an average content of 11.0 g/L (Fig. 6a). After mining, the whole area showed a downward trend. The K^+^ content changed to 8.2–9.8 g/L with an average content of 9.0 g/L, a drop of 18.2% (Fig. 6b). In 2009, the K^+^ content of horizon W_2_ was 4.3–13.1 g/L (average = 10.8 g/L). The high-value area was distributed in the south–central and northern parts of the mining area (Fig. 6c). After mining, the K^+^ content changed to 8.3–10.3 g/L (average = 9.4 g/L), an average reduction of 13%, especially in the northern and southeastern parts of the mining area (Fig. 6d). In 2009, the spatial distribution of K^+^ in W_3_ was similar to W_2_. The K^+^ content varied from 7.7 to 12.3 g/L with an average of 10.9 g/L (Fig. 6e). The K^+^ content after mining ranged from 6.5 to 9.3 g/L (average = 8.2 g/L), which represented a drop of 24.8%; the largest decline was in the north and southeast (Fig. 6f).

### Xinqing platform

**Figure 7:**
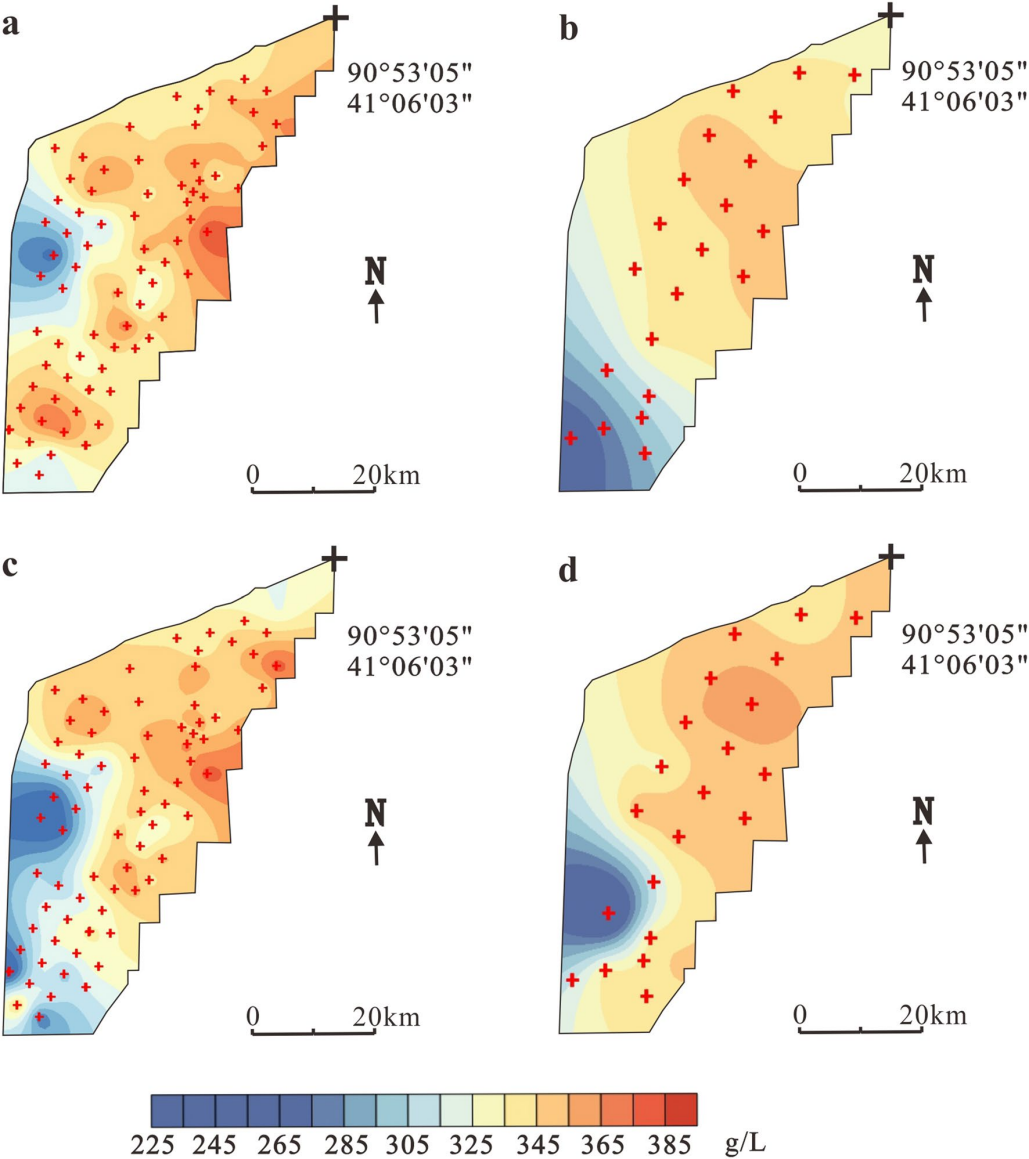
Temporal and spatial variations in salinity of different brine layers in Xinqing platform. (**a**) Layer W_2_ in 2010, (**b**) Layer W_2_ in 2017, (**c**) Layer W_3_ in 2010 and (**d**) Layer W_3_ in 2017. The red crosses indicate observation holes locations.

The study revealed two layers of potassium-rich brine ore, both of which are confined water layers. General exploration was carried out in August 2010, which reported a KCl (122b + 332 + 333) specific field reserve of 7.60 million tons in elevations ranging from 742 to 800 m^17^. The burial depth of the ore layer W_2_, W_3_ was about 10–20 m, 11.3–38 m respectively. Salinity comparisons in 2017 are shown in Fig. 7.

**Figure 8:**
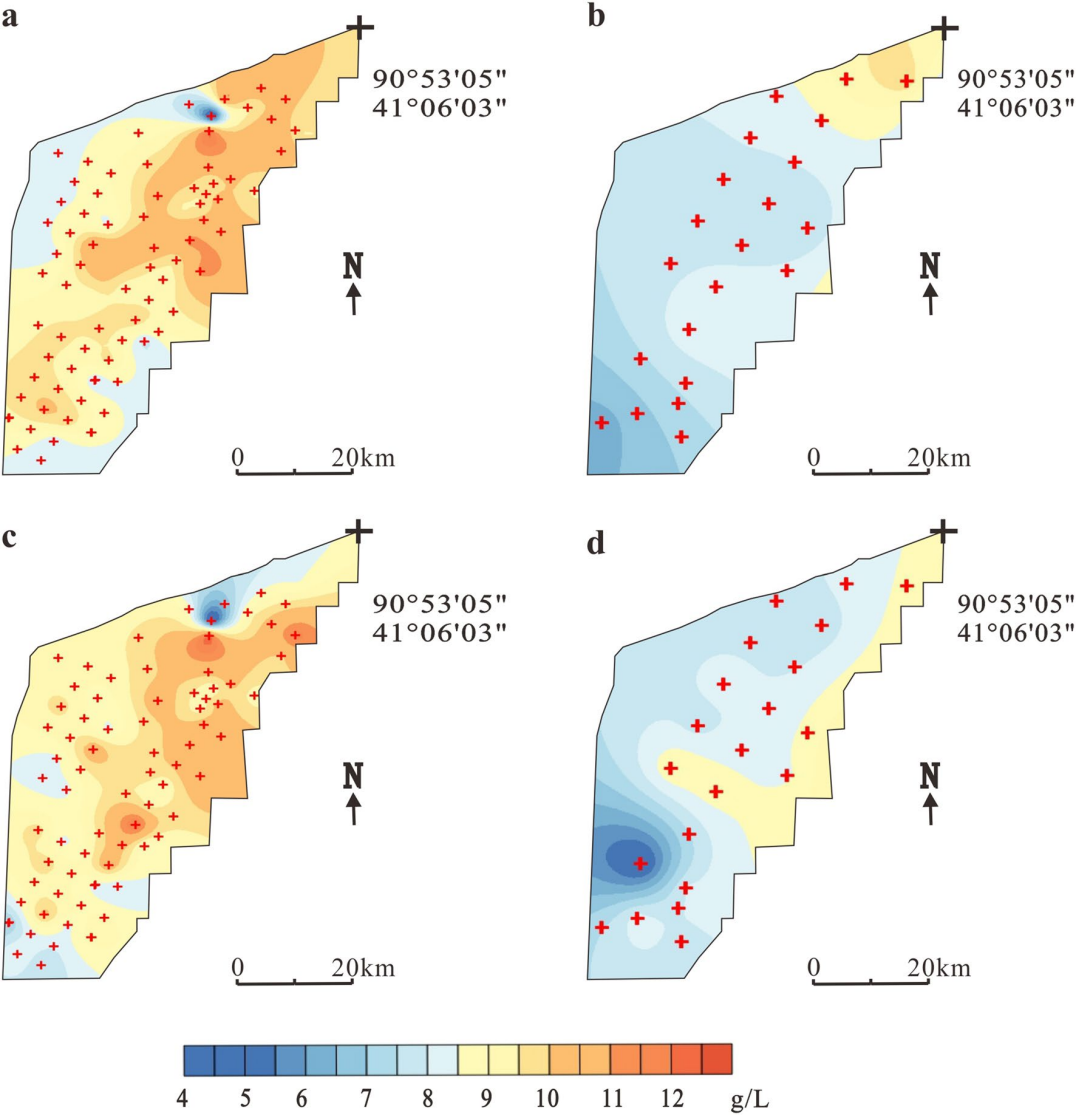
Temporal and spatial variations in K + content of different brine layers in Xinqing platform. (**a**) Layer W_2_ in 2010, (**b**) Layer W_2_ in 2017, (**c**) Layer W_3_ in 2010 and (**d**) Layer W_3_ in 2017. The red crosses indicate observation holes locations.

In 2010, the salinity of W_2_ in Xinqing platform was 268.6–374.0 g/L and the average value was 345.9 g/L^17^ (Fig. 7a). The high-salinity area is mainly located in the middle and eastern area, while the low-salinity area is located in the west and results from the lateral recharge of the Tarim River delta. After 7 years of mining, the whole mining area was desalinated and the salinity changed to 258.7–351.0 g/L, particularly in the southeastern part (Fig. 7b). The changes in the W_3_ layer before and after mining are consistent with W_2_. The mineralization degree of W_3_ in 2010 was 251.6–381.7 g/L (average = 333.5 g/L). After mining, the salinity decreased to 226.1–366.1 g/L. The high-value area moved to the northeast and the southeastern area faded significantly (Fig. 7c, d). At the same time, the K^+^ content also changed accordingly. The spatial distribution of K^+^ in 2010 and 2017 is shown in Fig. 8.

The K^+^ content of W_2_ in 2010 was 4.8–12.4 g/L and the average content was 9.8 g/L (Fig. 8a). The area of high K^+^ content is mainly located in the north–central region of Xinqing platform. In the northern mining area, there were very few samples with the low values, which may have been caused by the replenishment of bedrock fissure water of the Kuluktag. After 7 years of mining, the K^+^ content decreased significantly; the K^+^ content was 6.3–10.0 g/L (average = 8.1 g/L), which represents an average drop of 17.4%. The northeastern area was the only region to exhibit high values, while most of the western, central and southern regions were significantly diluted (Fig. 8b).

Figure 8c shows the spatial distribution characteristics of the K^+^ content in W_3_ in 2010. Similar to the distribution of W_2_, the K^+^ content varied from 4.5 to 12.5 g/L (average = 9.4 g/L). After 7 years of mining, the K^+^ content decreased by16% to 4.4–9.0 g/L (average = 7.9 g/L). The K^+^ content of the whole mining area decreased significantly, especially in the southern part, with declines as high as 50% (Fig. 8d).

### Underground brine drawdown

**Figure 9:**
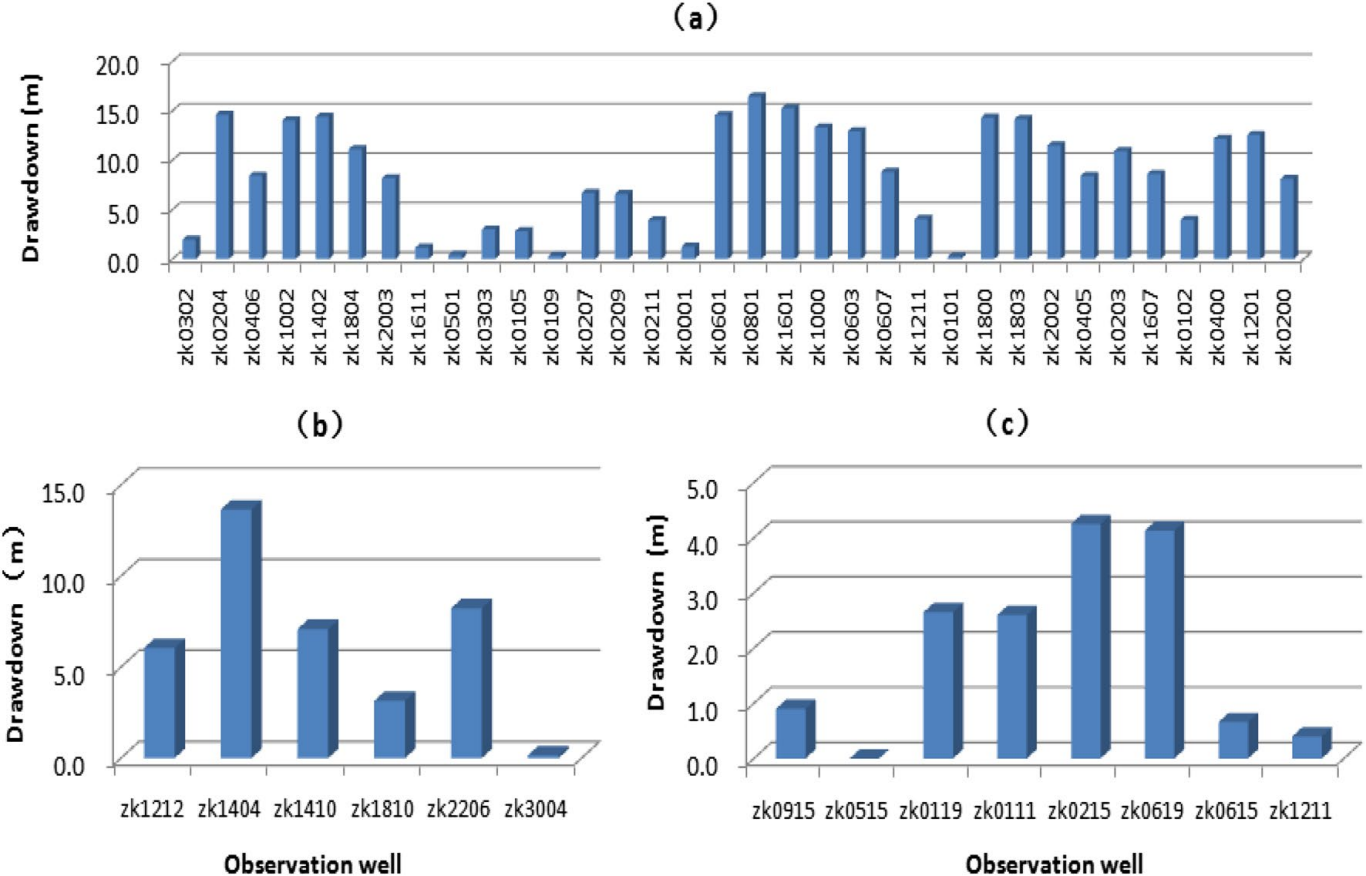
The drawdown of potassium-rich brine deposits in Lop Nor basin. (**a**) The depth of brine drawdown in Luobei depression. (**b**) The depth of brine drawdown in Tenglong mining area, (**c**) The depth of brine drawdown in Xinqing platform. The abscissa is the observation well number.

To describe variations in the underground brine table before and after large-scale mining of potassium-rich brine deposits in Lop Nor basin, this study used early monitoring data from three sub-mine areas. The data showed that the thickness of reservoir W_1_ in the Luobei depression was about 15–25 m in 2006. The burial depth was generally 1.7–2.3 m with a maximum of 4.5 m^15^. After mining, the brine table dropped significantly. From 2006 to 2017, the maximum brine drawdown reached 16.5 m (minimum = 0.2 m, average = 8.5 m; Fig. 9a).

The Tenglong mining area also experienced a large drop in the water table. In 2009, the average thickness of reservoir W_1_ in the northern part of the Tenglong mining area was 30–50 m, and the burial depth was about 0.7–4.0 m^16^. The 2017 monitoring data showed that the maximum drawdown was 13.6 m and the minimum drawdown was 0.2 m (average = 6.4 m; Fig. 9b).

There is no phreatic brine in the Xinqing platform. The shallowest confined brine layer is W_2_. In 2010, the W_2_ ore body was buried at a depth of 10–20 m with a thickness of 2–5 m^17^. After 7 years of mining, the reduction was small compared with a phreatic brine layer; the maximum drawdown was 4.3 m, the minimum was 0 and the average was 1.9 m (Fig. 9c).

**Figure 10:**
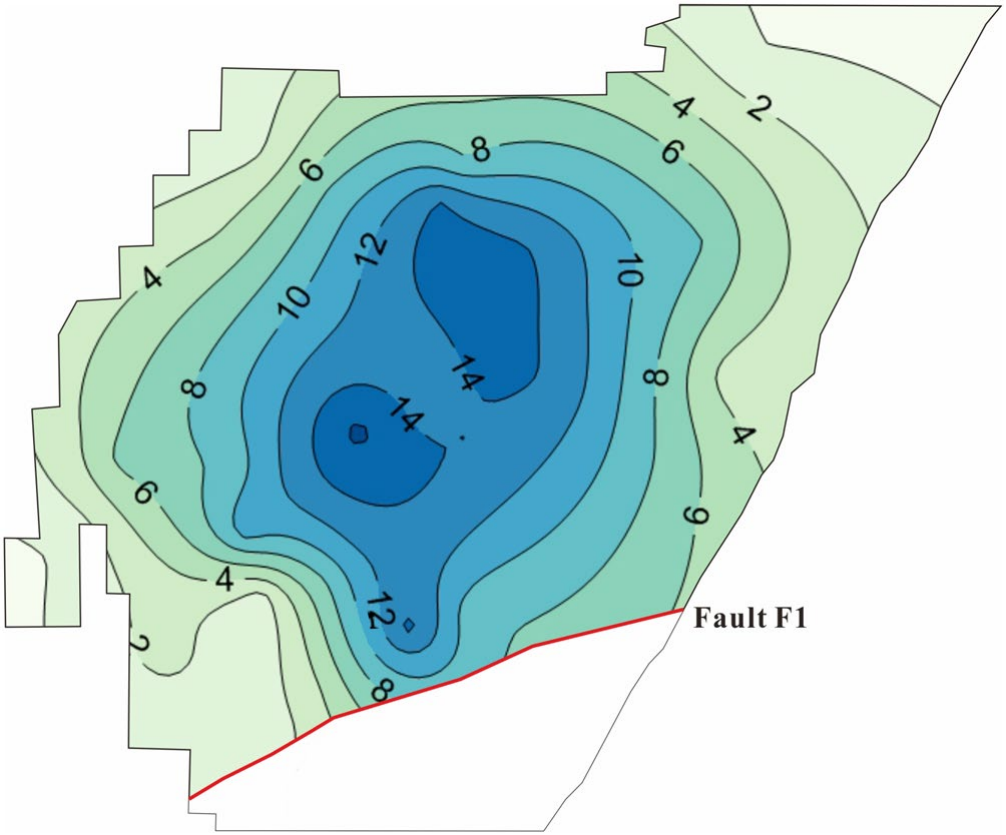
The drawdown distribution of potassium-rich brine layer W_1_ in Luobei depression and Tenglong mining area. The unit of brine drawdown is meter. The red line indicates the fault.

In general, the Luobei depression had the largest decline of the underground brine table and the drawdown of the water level in the most areas was greater than 10 m. Additionally, a settlement funnel was formed, centered on Luobei depression (Fig. 10).

In summary, the factors controlling the brine table drawdown include recharge (lateral subsurface recharge around the basin, atmospheric precipitation, condensate, and possible recharge from deep source) and discharge (artifical exploitation and evaporation). When the discharge is greater than the recharge, the groundwater table will continue to drop. However, the variations of salinity and potassium content are more complicated. It is not only controlled by the balance of recharge and discharge, but also involves the salt dissolution from evaporate strata and solute circulation in new flow field. Nevertheless, the artificial exploitation is still the main factor to control the variations in salinity and potassium content. It is estimated that the amount of underground brine extracted is ~ 29,600 × 10^4^ m^3^/a, and the total amount of recharge is only ~ 6700 × 10^4^ m^3^/a in the Lop Nor playa^15^.The difference between extraction and recharge is about 22,900 × 10^4^ m^3^/a. If the brine salinity is about 300 g/L (0.3 ton/ m^3^), it is estimated that more than 6500 × 10^4^ tons of salt were exported every year. Therefore, the brine table, salinity and potassium content have decreased significantly after more than 10 years of exploitation.

## Conclusions

China is a large agricultural country with a potash deficiency. At present, the self-sufficiency rate of potash fertilizer is only 50%. The Quaternary Lop Nor playa is one of the most important potash deposits in China. Although its mining history is not long, it supplies 50% of the Chinese potassium sulfate market. After more than 10 years of mining, the underground brine table and the grade of potassium sulfate in each sub-mine of the Lop Nor potash deposit have declined to varying degrees.

After large-scale mining, the K^+^ content in the Tenglong mining area decreased the most, followed by Xinqing and then Luobei. The average K^+^ content of horizon W_1_ was 9.0 g/L in the Tenglong area, which represented an average decrease of 18.2%. The average K^+^ in W_2_ was 9.4 g/L, indicating a decrease of 13%, and the average K^+^ content in W_3_ was 8.2 g/L (24.8% decline). After mining in the Xinqing mining area, the average K^+^ content in layer W_2_ was 8.1 g/L, with an average decline of 17.4%. The average K^+^ content in layer W_3_ was 7.9 g/L with a decline of 16.0%; in the southern part of the mining area, the decline was as high as 50%. After mining in Luobei depression, the average K^+^ content of W_1_ was 9.0 g/L, showing an average reduction of 4.3%. The average K^+^ content in W_2_ was 9.1 g/L (4.2% decline) and 9.5 g/L (3.1% decline) in W_3_.

The long-term monitoring data of the underground brine table from observation holes showed that the brine table of the Luobei depression obviously decreased after mining, with a maximum drawdown of W_1_ brine reaching 16.5 m (average = 8.5 m). A large drop in the brine level also occurred in the Tenglong mining area where the maximum drawdown of W_1_ was 13.6 m (average = 6.4 m). Xinqing platform did not contain phreatic brine. Therefore, compared with a phreatic brine layer, the resulting reduction was small and the lowest, shallowest confined layer (W_2_) had a relatively small reduction (maximum drawdown = 4.3 m, average = 1.9 m).

The artificial exploitation is the main factor to control the brine table drawdown, the variations in salinity and potassium content. According to the existing exploitation scale (~ 23,000 × 10^4^ m^3^/a), the service life of phreatic brine layer W1 is about 10 years. The resource reserves of confined brine body have not been identified, and further exploration is quite necessary to ensure the long-term sustainable utilization of the potassium-rich brines resources in the Lop Nor basin.
